# Whole genome SNP discovery and analysis of genetic diversity in Turkey (*Meleagris gallopavo*)

**DOI:** 10.1186/1471-2164-13-391

**Published:** 2012-08-14

**Authors:** Muhammad L Aslam, John WM Bastiaansen, Martin G Elferink, Hendrik-Jan Megens, Richard PMA Crooijmans, Le Ann Blomberg, Robert C Fleischer, Curtis P Van Tassell, Tad S Sonstegard, Steven G Schroeder, Martien AM Groenen, Julie A Long

**Affiliations:** 1 Animal Breeding and Genomics Centre, Wageningen University, De Elst 1, 6708WD, Wageningen, The Netherlands; 2 Animal Biosciences and Biotechnology Laboratory, Animal and Natural Resources Institute, Beltsville Agricultural Research Center, United States Department of Agriculture, Beltsville, MD 20705, USA; 3 Center for Conservation and Evolutionary Genetics, Smithsonian Conservation Biology Institute, Washington, DC 20013, USA; 4 Bovine Functional Genomics Laboratory, Animal and Natural Resources Institute, Beltsville Agricultural Research Center, United States Department of Agriculture, Beltsville, MD 20705, USA

## Abstract

**Background:**

The turkey (*Meleagris gallopavo*) is an important agricultural species and the second largest contributor to the world’s poultry meat production. Genetic improvement is attributed largely to selective breeding programs that rely on highly heritable phenotypic traits, such as body size and breast muscle development. Commercial breeding with small effective population sizes and epistasis can result in loss of genetic diversity, which in turn can lead to reduced individual fitness and reduced response to selection. The presence of genomic diversity in domestic livestock species therefore, is of great importance and a prerequisite for rapid and accurate genetic improvement of selected breeds in various environments, as well as to facilitate rapid adaptation to potential changes in breeding goals. Genomic selection requires a large number of genetic markers such as e.g. single nucleotide polymorphisms (SNPs) the most abundant source of genetic variation within the genome.

**Results:**

Alignment of next generation sequencing data of 32 individual turkeys from different populations was used for the discovery of 5.49 million SNPs, which subsequently were used for the analysis of genetic diversity among the different populations. All of the commercial lines branched from a single node relative to the heritage varieties and the South Mexican turkey population. Heterozygosity of all individuals from the different turkey populations ranged from 0.17-2.73 SNPs/Kb, while heterozygosity of populations ranged from 0.73-1.64 SNPs/Kb. The average frequency of heterozygous SNPs in individual turkeys was 1.07 SNPs/Kb. Five genomic regions with very low nucleotide variation were identified in domestic turkeys that showed state of fixation towards alleles different than wild alleles.

**Conclusion:**

The turkey genome is much less diverse with a relatively low frequency of heterozygous SNPs as compared to other livestock species like chicken and pig. The whole genome SNP discovery study in turkey resulted in the detection of 5.49 million putative SNPs compared to the reference genome. All commercial lines appear to share a common origin. Presence of different alleles/haplotypes in the SM population highlights that specific haplotypes have been selected in the modern domesticated turkey.

## Background

All commercial turkey lines descend from the South Mexican turkey (*Meleagris gallopavo gallopavo)* indigenous to Mexico, first domesticated in 800 BC [1]. In the US, the turkey is registered as a single breed with eight different varieties as defined primarily by plumage colour. Five of these eight varieties (Bronze, Narragansett, White Holland, Black and Slate) were registered in 1874, while the remaining three (Beltsville Small White, Bourbon Red, and Royal Palm) were registered in 1951, 1909, and 1971 respectively. There are a total of five wild turkey subspecies in North America but none of them contributed to the development of modern commercial lines [1].

Turkey is the second largest contributor of poultry meat consumed worldwide [2]. The production per bird doubled between 1970 and 2008 [2], largely due to selection pressure by the primary breeders for specific economically important traits, such as body weight, meat quality, and egg production [3-6]. Historically, quantitative genetics-based selection has been the primary strategy of genetic improvement of livestock [7]. This genetic improvement was largely applied to highly heritable traits, such as body size and breast muscle development. Genetic improvement of farm animals through selection may have increased production but has also resulted in a loss of genetic diversity [8]. The efficiency of these classical methods used for genetic improvement decreases when applied to traits that are difficult to measure or have lower heritability [7]. The availability of genome-based selection, based on a large number of SNPs at a density equivalent to the resolution of linkage disequilibrium (LD), has the potential to transform breeding and incorporate previously unavailable genetic information into commercial lines [9] which can be expected to change the impact of commercial breeding on diversity. A tremendous loss of SNP genetic diversity has been observed in chicken with significant absence of rare alleles (50% or more) in commercial breeds compared to ancestral breeds [10].

SNPs are a good marker type to study diversity. SNPs represent the most abundant source of genetic variation within the genome and are linked to heritable differences between individuals [11]. In addition, SNPs have a low mutation rate and are thought to be good genetic markers of potential disease phenotypes as well as for other complex traits [12]. Moreover, SNP markers are amenable to high throughput genotyping platforms and are valuable for a variety of genetic and genomic applications such as the construction of genetic and physical maps and the analysis of genetic diversity [13]. Next generation sequencing (NGS) has proven to be very effective for the large scale, genome-wide discovery of this type of genetic variation [14,15]. When a high quality reference genome sequence is available, genomic sequences of individuals can be aligned more easily to this reference genome to detect nucleotide variation [15,16]. Different studies have applied NGS platforms to achieve highly redundant coverage of the genome, a prerequisite for high quality genome-wide SNP discovery in the complex genomes of plants and animals [17-20].

Turkey genome assembly is based on commercial turkey, containing 39 autosomes and 2 sex chromosomes [21]. The most recent build, UMD 2.01, covers 90% of the genome [22] The size of the turkey genome assembly is 1.1 billion bases and, to date, about 600,000 SNPs [15,21] have been identified within the reference genome assembly. Increasing the number of SNPs identified in the turkey is an essential step for future improvement of economically important traits through genetic association studies [23-25].

Domestication of livestock species and a long history of migrations, selection and adaptation has created an enormous variety in breeds in livestock [8]. Phenotypic selection has created a wide diversity of breeds that are adopted to different climatic conditions and purposes [26]. Phenotypic variation observed between and among breeds of domestic animals is overwhelming compared with that in natural populations [26]. Chicken is considered the most closely related domesticated agricultural species to turkey. The observed phenotypic diversity in chicken is much larger than that of turkey, [26,27] most likely reflecting a much larger effective population size of chicken, before specialized commercial populations were established during the twentieth century. This is consistent with the extensive sequence diversity present in domestic chicken (5 SNPs/Kb) [28,29].

The presence of genetic diversity in domestic livestock species is of great importance for sustained genetic improvement of selected breeds in various environments, as well as to facilitate rapid adaptation to potential changes in breeding goals [30,31]. In animal breeding, crosses with non-commercial populations are rarely applied and genetically improved animals are often kept in small, closed populations. Small effective population sizes and epistasis can result in loss of genetic diversity, which can lead to reduced individual fitness and reduced response to selection [32,33]. Several studies have assessed genetic diversity in different livestock species [32,34-40] using different types of markers. A number of genetic diversity studies in chicken have reported loss of genetic diversity in commercial chicken populations because of high selection pressure and low effective population size [35,37,41]. A few studies have been published that explored genetic diversity in turkey genetic resources. However, these studies used a limited number of molecular markers [42,43] and only one study has been published that used 9 SNPs along with other molecular markers [44].

The goal of this project was to investigate turkey genome variation and to provide a resource for subsequent genomic work in the turkey and to cover a wide sampling of population for the development of a high-density SNP chip with minimal ascertainment bias. The SNP information will enable or improve application of genomic selection as well as association studies. We have used the identified SNPs to estimate relatedness among the sequenced turkey populations, which will uncover the genetic diversity available to breeders. Information of genetic diversity can be used in the design of breeding programs including making decisions on crosses between lines or introgression of genes from other commercial lines that may affect economically important traits such as growth, meat quality, fitness, and survival traits.

## Methods

### Populations

Eleven turkey populations were available for this study. Males from seven commercial lines, three heritage varieties and 113 years old samples of wild turkeys from South Mexico (SM turkeys) were used for whole genome sequencing. The seven commercial lines, L1 through L7, were obtained from two different primary breeding companies. The three heritage varieties were the Beltsville Small White (BvSW), the Royal Palm (RP) and the Narragansett (Nset) [45-47]. Tissue samples representing the wild population were obtained from the Bird Collection of the Smithsonian Institution’s National Museum of Natural History (USNM 165490, USNM 166330, and USNM 166329), and were originally collected in 1899 from Chihuahua, Mexico. These samples represent the progenitor subspecies, the South Mexican (SM) turkey. In total 32 individuals were selected for whole genome re-sequencing, with three males per population except for RP, which was represented by 2 males.

### Genomic DNA extraction, library preparation and sequencing

Considering mature erythrocytes in poultry are nucleated, genomic DNA was extracted from whole blood of the commercial and heritage lines with the QIAamp DNA blood Midi Kit (Qiagen, Valencia, CA); the procedure included a proteinase K digestion followed by column purification. Integrity of high molecular weight DNA following the extraction was confirmed by agarose gel analysis. Genomic DNA was sheared using the Covaris S2 to yield an average fragment size of 450 bp, as determined with the Agilent Bioanalyzer 2100 (Agilent, Santa Clara, CA). The DNA from the three historic SM samples was extracted from the toe-pads in the ancient DNA laboratory at the Smithsonian Institution’s Center for Conservation and Evolutionary Genetics, that is fully equipped to avoid contamination with modern DNA. DNA extraction followed a standard protocol of proteinase k and DTT digestion followed by phenol-chloroform extraction and centrifugal dialysis with Centricon concentrators (following methods provided in [48]). An extraction blank sample was used as a no-sample control in each round of extraction. Extractions involved alternation of turkey samples with samples from other avian or non-avian taxa, in order to detect potential cross-contamination among extracts. Extracts of the samples and extract controls were subjected to PCR with standard avian mtDNA primer sets (Cytochrome *b*, ND2; [49]) followed by sequencing of positive products to confirm the isolation of turkey DNA from the toe pads. The genomic DNA of the SM samples ranged from 40-43 bp (Agilent Bioanalyzer).

Genomic libraries were prepared with the Paired-end Sequencing Sample Preparation Kit (Illumina, San Diego, CA) with 5 μg of genomic DNA for commercial and heritage lines according to the manufacturer’s instructions; for the SM samples 0.54 μg was used to construct the libraries. All genomic DNA libraries were validated with the Agilent Bioanalyzer (model 2100). The automated cBot Cluster Generation System (Illumina) was used to generate clusters on the flow cell. Each individual was sequenced (paired-end; read length 120 bp) in a single lane of a flow cell using the Illumina GAIIx. The DNA extracted from museum samples for the SM turkeys was highly degraded, and thus single-end reads of 40 bp were generated from these samples.

### Sequence mapping and SNP identification

Sequence reads of each individual from the domesticated populations (heritage varieties and commercial lines) were filtered on base quality; reads were trimmed if three consecutive bases had an average Phred-like quality score of less than 13. Both sequences in a pair needed to exceed 40 bp in length after trimming to be retained for analyses. Sequence reads from the individuals of the SM population were not quality-trimmed before further analyses since they were sequenced to a length of 40 bp only. Sequence reads were aligned against the turkey reference genome (UMD 2.01) using the MOSAIK aligner [50]. Mapping of reads from each individual to the reference genome sequence was performed with hash size 15 (hs), 100 maximum hash positions (mhp), an alignment candidate threshold (act) of 20, and a maximum mismatch percentage (mmp) of 5. Banded Smith-Waterman algorithm (bw = 41) was used to increase the speed of alignments. The algorithm implemented in MOSAIK calculates a mapping quality for each sequence and measures the probability that a sequence belongs to a specific target. The alignments were sorted using MosaikSort. Finally, the file was converted to BAM format using MosaikText. All BAM files have been uploaded to NCBI's Sequence Read Archive (SRA) database under the study accession number “SRP012021.2”.

The mpileup function of SamTools version 0.1.12a [51] was used to call variants, separately for each turkey population. The view option of bcftools [51] was used to call the genotype at each variant for each animal. Genotypes were called for each animal with a minimum genotype quality of 20, and a read depth between 1 and 25. At least one individual in a population needed to have a genotype call that met these criteria at a particular position. A SNP that passed the above mentioned criteria were considered as a putative SNP. Putative SNPs were categorized into fixed differences compared to the reference genome and segregating SNPs. Homozygous non-reference genotypes that were the same in all individuals of a population were considered fixed SNPs, while the SNPs that had variable/heterozygous genotypes in a population were considered segregating SNPs.

To estimate heterozygosity (heterozygous SNPs/kb), mpileup genotyping analysis (described above) was used and the number of heterozygous SNPs was calculated at the reference bases covered from 5 to 10 fold. For each individual in a population, heterozygosity was estimated by dividing the total number of discovered heterozygous SNPs by the total genome sequence covered from 5 to 10 fold. Population heterozygosity was estimated by averaging the heterozygosity of all individuals within a population.

### Functional annotation of SNPs

The gene-based analysis of ANNOVAR software [52] was used to functionally annotate the putative SNPs. For each putative SNP, the location (exonic, intronic, intergenic, 5’UTR, 3’UTR, splice acceptor or donor site, downstream or upstream) and the functional annotation (nonsynonymous, synonymous, stop codon gain or loss, and amino acid changes) were determined based on the turkey reference genome (UMD 2.01). Gene annotations used in this analysis were taken from Ensembl [53]. Standard settings for gene based analysis of ANNOVAR were used.

### Nucleotide diversity and false discovery rate

Genome wide mapping density, or read depth distribution, and the nucleotide diversity across the whole genome were assessed for each individual of the 11 turkey populations. Read depth distribution was used to calculate average sequence coverage across the whole genome. To get genotypes of each individual without imputation, pileup function of SamTools version 0.1.12a [51] was used for the estimation of nucleotide diversity across the whole genome. Genotypes were called for each individual using minimum genotype quality of 20, and a read depth between 3 and 15. The number of heterozygous and homozygous non-reference SNP calls was estimated compared to the reference genome within a 300 Kb window. In order to estimate SNP false discovery rate (FDR), 30 large genomic regions of variable sizes (ranging from 2.7-10.5 Mb on variable positions at chromosomes 1, 3 and 10) were investigated where one individual from each of the 10 domesticated populations was clearly homozygous for a single haplotype. Homozygous regions were identified by visual inspection of the nucleotide diversity plots for turkey chromosome 1, 3 and 10. Any SNP within these regions were considered to be false positives. The false discovery rate was calculated as the total number of heterozygous SNP positions divided by the total number of bases covered (1–25 fold coverage) in these 30 regions.

### Genetic diversity analysis

PHYLIP software, version 3.69 [54] was used to calculate pairwise Nei’s genetic distance [55] among all the individuals from the 11 turkey populations. SNPs for which genotypes were called in at least 9 turkey populations (irrespective of whether SNPs were segregating in all these populations) were selected and utilized for the genetic diversity analysis. Threshold of at least 9 turkey population was selected to increase number of selected SNPs for analysis and to make sure presence of selected SNPs in maximum populations to have a reliable genetic comparison. Pairwise genetic distance analyses were based on marker data that the individuals had in common, because PHYLIP is unable to deal with missing data [36]. Mega 5.0 [56] was used for hierarchical clustering using a Neighbour-joining procedure on the genetic distance matrix for all the individuals. The wild population was used to root the phylogenetic tree.

### Non-reference allelic state

The genome of each individual was screened, using the nucleotide diversity analysis described above, for the occurrence of non-reference allelic states. Determining the ancestral allelic state of SNPs was not possible because species with appropriate evolutionary distance are not available. Chicken is considered a closely related domesticated agricultural species to turkey but the evolutionary distance to the last common ancestor of these two species is around 30 million years [57]. To quantify regional changes in genomic diversity between SM and the domesticated populations, we used heterozygosity as well as the presence of non-reference allelic homozygosity of the positions sufficiently covered by sequencing.

The difference in non-reference allele homozygosity between domesticated and the SM turkey populations was calculated for each bin. This difference was then divided by the average homozygous non-reference allele SNP density for the bin to yield a relative measure that can be compared between bins with different levels of variation.

The ratio of non-reference homozygosity in wild SM vs. domesticated populations was calculated within bin sizes of 300 Kb. A high ratio points to non-reference alleles being lost, or decreased in frequency during domestication and selection. A high ratio of non-reference homozygosity, in combination with low heterozygosity in the domesticated populations, is interpreted as a reduction of allelic variation from wild to domesticated populations, or “fixation of the reference alleles”. A bin was considered “fixed for the reference allelic state” in domesticated populations when two conditions were met. First, bins were considered “fixed” when heterozygosity was equal or lower than 0.0002 on average across all domesticated populations. This threshold was chosen because only 5% of the bins had a heterozysity equal or lower than 0.0002 (1 heterozygous position/5000 bp). Second, bins that were considered “fixed” had to have a ratio of non-reference allele homozygosity above or equal to 1.73, which means that the non-reference allele homozygosity of the wild population must be at least 73% higher than the domesticated populations. This threshold was chosen because only 5% of all the bins in the genome had a ratio equal or higher than 1.73.

### Ethical approval for the use of animals in this study

Although animals were used in this study, no direct experiments were performed on them. Blood sample collection was carried out by highly skilled and experienced personnel from the breeding companies. No approval from the ethics committee was necessary according to local legislation.

## Results

### Whole-genome resequencing and SNPs discovery

The obtained sequence from the DNA samples of the domestic populations (heritage varieties and the commercial lines) varied from 2.30-13.21 Gbp (Giga basepairs) per individual. After quality trimming and alignment of the short reads, the percentage of bases in the reference genome covered by at least 1 and a maximum of 25 reads varied from 47.48% to 86.13% for the animals analyzed (Table 1). The sequences generated from SM turkeys varied from 0.41-0.82 Gb of sequence per individual. The sequence depth at bases covered by at least one read ranged from 1.38 to 1.81 for the SM samples and 2.07 to 6.72 for the domesticated turkey lines (Table 1).

**Table 1:** Alignment statistics for the individuals from different turkey populations

**IDs**	**Sequence coverage (fold)**^**1**^	**Assembly coverage (%)**^**2**^	**Assembly coverage 1-25X (%)**^**3**^
**L1a**	5.12	79.04	78.93
**L1b**	4.72	83.88	84.04
**L1c**	5.61	84.10	83.85
**L2a**	6.54	85.91	85.85
**L2b**	6.72	86.19	86.13
**L2c**	5.18	80.16	80.05
**L3a**	6.32	85.98	85.68
**L3b**	5.75	85.26	85.21
**L3c**	6.24	85.91	85.72
**L4a**	6.19	85.58	85.51
**L4b**	5.75	84.65	84.58
**L4c**	5.13	84.14	84.12
**L5a**	3.52	71.18	71.14
**L5b**	5.18	71.35	71.27
**L5c**	5.73	68.35	68.08
**L6a**	2.88	65.14	65.13
**L6b**	4.50	77.53	77.49
**L6c**	4.52	81.45	81.43
**L7a**	5.46	78.59	78.39
**L7b**	4.61	57.86	57.70
**L7c**	4.99	70.88	70.78
**BvSW1**	4.55	83.21	83.19
**BvSW2**	5.72	48.33	47.48
**BvSW3**	5.59	82.24	82.13
**Nset1**	2.07	53.84	53.82
**Nset2**	5.39	83.94	83.86
**Nset3**	5.17	79.42	79.29
**RP1**	5.31	60.31	60.05
**RP2**	5.00	63.54	63.43
**SM1**	1.81	47.10	47.06
**SM2**	1.38	29.32	29.30
**SM3**	1.73	45.41	45.40

^**1**^ Average sequence depth of each base in the reference genome that is covered by at least 1 read. The used turkey reference genome (UMD 2.01) has genome size of 1,061,982,190 bp, which is 90% of the total turkey genome size. ^**2**^ Percentage of reference genome that is covered by at least one read. ^**3**^ Percentage of reference genome that is covered by 1–25 reads.

In total, 5.49 million putative SNPs were identified compared to the reference genome (Table 2). Of these 5.49 million SNPs, 4.76 million SNPs were segregating in at least one population (Table 2). The number of segregating SNPs for the different turkey populations varied from 0.12 to 1.58 million, with the highest number of segregating SNPs observed in L3 and the lowest number observed in SM (Table 3). The lowest number of fixed SNPs was observed in L3 and the highest number of fixed SNPs was observed in BvSW (Table 3). The transition to transversion (Ti/Tv) ratio of the SNPs discovered is 2.45. Of the total 5.49 million SNPs discovered, 75,254 were located in exonic regions, including 23,795 nonsynonymous , 52,506 synonymous, 377 stop gain and 8 stop loss variants. The majority of these exonic SNPs, 66,795 or 89% were segregating within the populations analyzed (Table 4).

**Table 2:** Heterozygosity and the number of SNP observed in each individual of different turkey populations

**IDs**	**Homozygous NR SNP**^**1**^	**Heterozygous SNP**	**Heterozygous SNP 5-10X**	**Genome covered 5-10X (bp)**	**Heterozygosity Kb**^**-1**^
**L1a**	663,406	659,351	369,849	320,663,179	1.15
**L1b**	686,583	648,928	385,673	396,624,720	0.97
**L1c**	626,434	737,472	403,423	375,734,398	1.07
**L2a**	827,249	755,318	504,787	532,961,711	0.95
**L2b**	896,728	757,226	514,059	554,379,839	0.93
**L2c**	869,872	562,653	311,525	329,283,144	0.95
**L3a**	568,439	762,252	519,228	532,049,588	0.98
**L3b**	434,157	427,393	567,558	527,841,728	0.99
**L3c**	608,276	834,241	164,167	166,315,925	1.08
**L4a**	720,530	616,567	440,086	454,905,713	0.80
**L4b**	760,762	692,079	385,458	439,002,235	0.97
**L4c**	807,407	618,335	403,201	503,650,627	0.88
**L5a**	666,287	340,436	160,698	180,577,454	0.89
**L5b**	652,149	352,682	165,723	144,150,087	1.15
**L5c**	736,951	520,850	251,977	223,238,275	1.13
**L6a**	581,773	294,736	109,405	115,435,304	0.95
**L6b**	644,421	567,275	330,736	306,448,666	1.08
**L6c**	638,770	579,232	341,869	348,094,277	0.98
**L7a**	736,881	550,299	300,174	305,785,110	0.98
**L7b**	698,647	379,941	185,444	161,035,610	1.15
**L7c**	730,143	504,513	275,118	252,564,184	1.09
**BvSW1**	1,053,237	417,544	241,641	372,524,318	0.65
**BvSW2**	1,071,513	269,338	103,333	144,219,590	0.72
**BvSW3**	1,086,121	525,262	299,713	369,633,525	0.81
**Nset1**	643,308	79,232	25,217	144,546,998	0.17
**Nset2**	667,797	519,815	9,929	4,717,330	2.10
**Nset3**	773,183	804,627	454,052	320,395,210	1.42
**RP1**	885,734	510,427	154,899	167,716,001	0.92
**RP2**	842,442	522,599	276,752	208,702,070	1.33
**SM1**	551,149	69,199	11,106	9,379,558	1.18
**SM2**	551,380	17,275	2,030	744,899	2.73
**SM3**	551,543	44,784	6,921	6,868,381	1.01

^**1**^ Homozygous non reference SNPs observed in each individual.

**Table 3:** **Discovered segregating, and the fixed number of SNPs along with the observed heterozygosity Kb**^**-1 **^**in each turkey population**

**Population ID**	**Segregating SNPs**^**1**^	**Fixed SNPs**^**2**^	**Heterozygosity Kb**^**-1**^
**L1**	1,563,553	617,893	1.07
**L2**	1,504,682	781,352	0.94
**L3**	1,589,525	502,807	1.01
**L4**	1,441,173	709,507	0.88
**L5**	950,425	674,038	1.06
**L6**	1,139,459	613,069	1.00
**L7**	1,097,788	673,807	1.07
**BvSW**	926,733	1,047,010	0.73
**Nset**	1,194,570	708,773	1.23
**RP**	883,602	813,164	1.12
**SM**	120,305	552,032	1.64

^**1**^ The total number of SNPs detected compared to the reference genome in which the non-reference allele is segregating in a population. ^**2**^ The total number of SNPs detected compared to the reference genome in which only the non-reference allele is found in a population.

**Table 4:** Number of SNPs detected

**Variants**	**Reference total**^**1**^	**Segregating Total**^**2**^
Nonsynonymous	23,795	20,463
Synonymous	52,506	47,281
Stopgain	377	295
Stoplost	8	7
Exonic splice site	1,437	1,256
Exonic	75,254	66,795
Splice acceptor or donor site (interonic)	734	607
5'UTR/3'UTR	8,933	7,661
Upstream/downstream	142,829	124,005
Intronic	1,749,427	1,518,783
Intergenic	3,514,102	3,044,243
ncRNA	1,044	916
**Total**	**5,493,760**	**4,764,266**

^**1**^ SNPs detected compared to the reference genome in which the non-reference allele is detected in at least one of the 29 individuals. ^**2**^ Detected segregating SNPs within all turkey individuals.

### Heterozygosity

**Figure 1:**
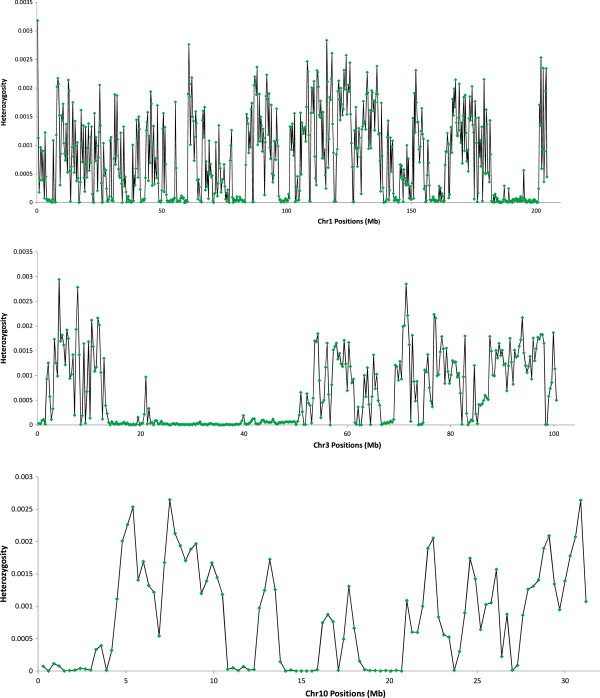
**Nucleotide diversity showing heterozygous and homozygous regions within chromosome 1, 3 and 10.** Heterozygosity across chromosome 1, 3 and 10 for individual L1c. Chromosome positions (Mb) are presented on the x-axis and on the y-axis heterozygosity is given as the density of heterozygous SNPs corrected for the number of bases covered within a window size of 300 Kb. Note the clear homozygous regions at 188–198 Mb for chromosome 1, 24–38 Mb for chromosome 3 and 18–21 Mb for chromosome 3.

The number of heterozygous genotypes detected within the individuals from the ten domesticated populations (heritage varieties and the commercial lines) varied from 0.08 to 0.80 million with an average of 0.55 million heterozygous genotypes per individual. Individuals from the SM population showed relatively low numbers of heterozygous SNPs; between 0.01 and 0.07 million. Heterozygosity (heterozygous SNPs/kb) of all individuals from the different turkey populations ranged from 0.17-2.73 while heterozygosity of populations ranged from 0.73-1.64 (Table 2 &3). The BvSW population had the lowest heterozygosity, while SM showed the highest heterozygosity within the analyzed populations (Table 3). Observed average nucleotide diversity in the 10 largest chromosomes was 0.0005 segregating SNPs per nucleotide position while average nucleotide diversity in the smaller chromosomes (20–30) was 0.0007. Chromosome Z showed the lowest nucleotide diversity with 0.0002 segregating SNPs per nucleotide position. Based on observed homozygous regions (Figure 1), interpreted to represent two copies of the same Identical By Descent (IBD) haplotype, the estimated average heterozygous genotype FDR was 0.00002 per nucleotide position in the reference genome (ranging from 0.000012**-**0.000023 in the different individuals).

### Genetic diversity

**Figure 2:**
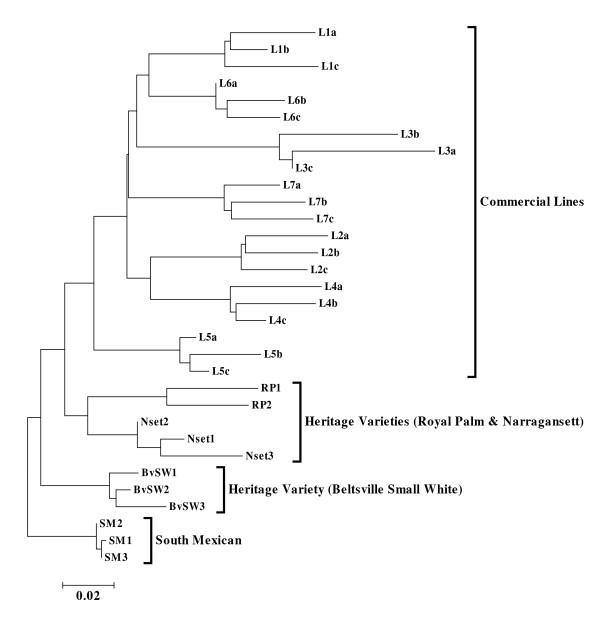
**Dendrogram for 32 individuals from 11 different turkey populations based on Nei’s genetic distance.** Individuals from the same population are clustered together and **i**nter-line comparisons demonstrate that commercial lines form a cluster distinct from heritage varieties.

There were 223,264 SNPs segregating in at least 9 turkey populations, and these were used to calculate Nei’s pair wise genetic distances. The tree based on Nei’s genetic distance for the 32 turkey individuals from the 11 different turkey populations presents their genetic relationships (Figure 2). Individuals from a specific turkey population clustered closely together. Inter-population comparisons demonstrated that commercial lines formed a cluster that was distinct from heritage lines with the exception of the L5 line, which exhibited a closer genetic relation to the heritage varieties. Among the heritage varieties, RP and Nset were more genetically related than either to BvSW. Individuals from the SM population also clustered together and showed relatively closer genetic relation with BvSW population.

### Non-reference allelic state

**Figure 3:**
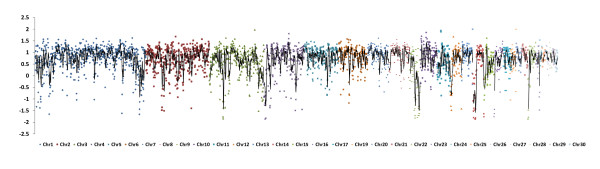
**Difference in non-reference allele homozygosity per nucleotide position between domesticated and the SM populations.** Y-axis denotes difference in non-reference allele density per nucleotide position relative to the mean level of variation discovered between domesticated and the wild SM turkey populations. Five turkey chromosomes 3, 4, 9, 14 and 22 shows visible difference in peaks of these chromosomes.

**Figure 4:**
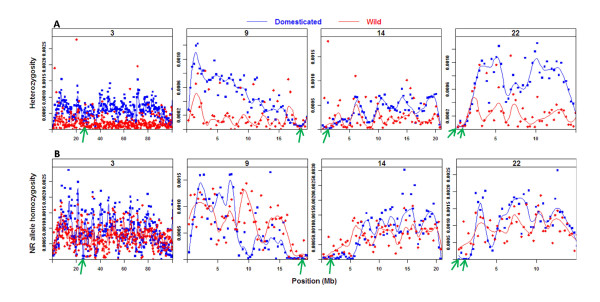
**Comparison of heterozygosity and the non-reference allele homozygosity between wild and domesticated turkeys.A**) Heterozygous SNP density per nucleotide position (y-axis) within a bin size of 300Kb, x-axis shows positions in million basepairs (Mb) for turkey chromosomes 3, 9, 14 and 22. **B**) Non-reference allele homozygosity per nucleotide position (y-axis) within a bin size of 300Kb, x-axis shows positions in million basepairs (Mb) for turkey chromosomes 3, 9, 14 and 22. Green arrows identify regions fixed for reference haplotype in domesticated populations.

Six regions on five different turkey chromosomes (3, 4, 9, 14, and 22) showed differences between the SM and the domesticated populations with respect to the occurrence of no-reference wild type and the reference allelic states (Figure 3). Domesticated populations predominantly showed the reference allelic state, while the SM populations predominantly showed the no-reference wild type allelic state within these regions. These six regions were then examined with respect to the heterozygous SNP density per nucleotide positions within the same bin size. Within these six regions, nucleotide diversity for all the domesticated populations was found to be close to zero, except for one region on chromosome 4 that showed high segregation of non-reference alleles within the domesticated populations (Figure 4). The other five genomic regions, two regions in chromosome 22 and one region in each of the remaining three chromosomes, (3, 9 and 14), met the criteria mentioned in the methodology section (Additional file 1). These genomic regions were considered fixed for the reference allelic state in the domesticated populations.

## Discussion

In this study, we performed whole genome sequencing for SNPs discovery and used the identified SNPs to characterize genetic diversity in the turkey genome. To avoid imputation of genotype calls across the different populations, mpileup was applied within each population separately because the applied method (mpileup) relies in part on Hardy-Weinberg Equilibrium (HWE) for imputation of genotypes [51].

By using a NGS (Illumina GAIIx) approach, we discovered millions of high quality SNPs in the turkey. Next generation sequencing approaches are considered highly reliable for genome-wide discovery of sequence variation [15], when used to compare different lines/strains to a reference genome [58]. The adoption of NGS platforms for the discovery of genomic variation has now become mainstream [15,58-60].

The high quality of the SNPs discovery reported here is reflected by the low FDR of 0.00002 per nucleotide in the genome. This FDR suggests around 2.1 x 10^4^ false discovered heterozygous positions per turkey genome (size of 1.1 x 10^9^ base pairs). The SNPs FDR rate for the same 10 animals from distinct turkey populations was estimated after correcting for the coverage and using estimates of FDR per nucleotide position. The SNPs FDR was found to be 2.6%, a number that is similar in magnitude as found previously in the human 1000 Genome Project. In addition to the low FDR, we found a transition/transversion (Ti/Tv) ratio within the expected range. The expected Ti/Tv ratio of true novel variants can vary with the targeted region (whole genome, exome, specific genes), species and also can vary greatly by the CpG and GC content of the region [59-61]. In the case of exomes, an increased presence of methylated cytosine in CpG dinucleotides in exonic regions leads to an increased Ti/Tv ratio [61] due to an easy deamination and transition of a methylated cytosine to a thymine [61]. It is also observed that GC content is higher in birds and mammals than in invertebrates [62]. Observed Ti/Tv ratio in our study of turkey is in concordance with the findings from Dalloul et al. [21], but slightly higher (2.45) than that of human. This higher ratio is most likely explained by the smaller genome size and a higher GC percentage in bird genomes.

We report the number of segregating as well as total number of SNPs with their functional annotation. The 23,795 nonsynonymous variants that were observed can potentially change the structure of proteins, possibly resulting in altered phenotypes [63]. Out of these nonsynonymous SNPs, 9,204 were unique to commercial population which may have been detected due to higher coverage and number of individuals for the commercial turkey population. We observed 5,417,069 SNPs that were present in non-protein coding DNA. Furthermore, we discovered 1,749,427 intronic variants, some of which may alter gene expression or result in alternative splicing [64,65]. Variants located in intergenic regions, such as promoter, enhancer and silencer regions can result in altered gene expression. The human genome comprises over 98% non-protein coding DNA [66]. Estimates suggest that at least 5.5% of the human genome, including 3.5% of its noncoding fraction, consists of regions under purifying natural selection against deleterious alleles [67-69]. In addition, most of the variants involved in complex genetic diseases in humans are not located in coding regions [59]. Likewise, variation outside of coding regions may be responsible for economically important traits in domesticated species, e.g. disease resistance, meat quality, efficient growth, or high egg production. The functional information of these variants can help in prediction of phenotypes or genetic merit with higher accuracy and selection of individuals can be done accordingly.

The estimated average frequency of 1.07 heterozygous SNPs Kb^-1^ in the turkey is substantially lower than in chicken, which was previously reported as 4.28 and 2.24 heterozygous SNPs Kb^-1^ in two different studies [28,29]. In our study, heterozygous SNP discovery was found to be affected by the sequence coverage (e.g. sequence coverage in L6a, Nset1 and the SM animals was low and as a result the number of observed heterozygous SNPs was also low). Estimates of heterozygosity were therefore obtained only from genomic regions that were covered 5 to 10X to adjust for the effect of low sequence coverage.

Modern commercial turkey lines are derived from historic turkey populations that displayed low variation as a result of small effective population size [70,71]. Heritage (Nset and RP) and the wild SM turkey populations showed higher heterozygosity compared to the commercial populations, which is concordant with the findings of previous studies on ancient and overexploited species [72-74]. The heritage variety BvSW showed the lowest heterozygosity of all turkey populations, which is consistent with the severe bottleneck that this population went through in 2000 (Alexandra Scupham, Personal communications).

Most birds have a characteristic division in chromosome size, with 5 or 6 large chromosomes, around 5 intermediate size chromosomes, and 25 to 30 very small chromosome pairs. In our study, we observed higher nucleotide diversity on smaller chromosomes compared to the larger turkey chromosomes which is in agreement with the previous study [75]. Since the recombination rate is far higher at the smaller sized turkey chromosomes as compared to large chromosomes [76], which leads to lower linkage disequilibrium and higher haplotype diversity on the smaller chromosomes [77]. Although the high gene-density of the smaller chromosomes would make them susceptible to hitchhiking effects that could erode genetic variation, hitchhiking effects appear to be offset by the far higher recombination rate of the micro-chromosomes. Chromosome Z showed the lowest nucleotide diversity, which is concordant with the findings of Dalloul et al. [21]. This low nucleotide diversity of chromosome Z is likely the result of a lower effective population size of this chromosome and lower recombination rate [78].

The presence of different allelic states in the wild SM and the domesticated populations is a demonstration of their divergence during the course of domestication event. Domesticated turkey lines were selected (artificially or naturally) for non-wild type alleles. Domestication has involved the selection on a desired trait(s) [79], and previous studies on domesticated animals have demonstrated selective pressures on genes related to growth [64] and coat colour [80,81]. Such studies have also demonstrated that artificial selection might have contributed to reduced polymorphism levels and increased LD in domesticated species [10,82-84]. On-going directional selection causes footprints of selection identifiable as regions where the derived allele frequency is higher than non-selected regions [29,85,86]. Most of the turkey chromosomes are acrocentric and the five genomic regions that were found to be fixed for the reference alleles within the domesticated populations seem to be located close to the centromere [87]. This may explain the presence of a strong hitchhiking effect due to the low recombination rate close to the centromeres. These fixed turkey genomic regions were then investigated for the presence of report QTLs corresponding to these regions. While QTLs were not found within the fixed regions [88], there were QTLs for growth and meat quality on chromosome 3, a QTL for percentage drip loss on chromosome 14 and a growth related QTL on the chromosome 22 [88]. These QTLs for different traits on chromosomes 3, 14 and 22 were located at distinct positions that did not coincide with the observed regions with high reference allele frequency. Due to the evidence of the presence of structural and functional conservation in the turkey and the chicken genomes [76,88] and also the limited availability of information on turkey QTLs, these 5 turkey genomic regions that were found to be fixed for reference alleles within domesticated populations, were aligned with the chicken genome sequence (WASHUC2) to determine the position of these turkey genomic regions within the chicken genome (Additional file 1). Regions of the chicken genome exhibiting synteny with turkey were then examined for the presence of known chicken QTLs [89]. Several QTL were identified within these 5 genomic regions (Additional file 1) and most were related to growth traits (Additional file 1). Production census of turkeys from the last few decades [2] show that turkeys are highly selected for growth and this high selection pressure might have favoured reference alleles in domesticated populations. Since several of the regions identified in this study are probably close to a centromere, the effect of selection may have extended over a larger region due to the likely reduced recombination rate in centromeric parts of the genome.

The genetic diversity analysis among the 11 different turkey lines showed that the heritage varieties and the commercial populations are derived from the wild South Mexican population. All of the heritage varieties (BvSW, RP and Nset) are closely related which is in agreement with previously published data [43,44]. The relatedness of these heritage varieties can probably be explained either by historic nature, a common origin, selection for similar traits/phenotype or a relatively low selection pressure in these varieties. The Nset, RP and BvSW heritage lines were developed in America in 1800, 1920 and 1930, respectively [70,71]. It is assumed that the colour pattern of RP is derived from crossbreeding with Narragansett and perhaps another variety, as Nset colour mutation is a component of the final RP colour (Smith et al., 2005). The close genetic relatedness observed between RP and Nset in our study is also concordant with that assumption and with previous studies [43,44]. According to Figure 2, commercial lines from different breeding companies did not resolve into two separate groups. The close relatedness of the L5 commercial line to the heritage lines is not surprising as it represents a female line selected for medium weight, conformation and egg production; selected traits characteristic of the heritage lines [71]. The other commercial lines that cluster separate from L5 in the dendrogram were selected for different objectives such as higher body weight and rapid growth.

## Conclusion

The turkey genome is much less diverse with a relatively low frequency of heterozygous SNPs as compared to other livestock species like chicken and pig. The whole genome SNP discovery study in turkey resulted in the detection of 5.49 million putative SNPs compared to the reference genome. All commercial lines appear to share a common origin. Presence of different alleles/haplotypes in the SM population highlights that specific haplotypes have been selected in the modern domesticated turkey.

## Competing interests

The authors declare that they have no competing interests.

## Authors’ contributions

MLA, JWMB, MGE and HJM analysed the data. LAB assisted and trained laboratory personnel in genomic DNA isolation from blood and preparation of all libraries. RCF isolated the museum specimen DNA. CPVT assisted with project design and coordination. TSS helped develop sequencing strategy and guided library preparation of ancient DNA samples. SGS was responsible for sequencing processing. JAL conceived and developed the project, organized blood sample collection and DNA sequencing, and was the Principal Investigator (Agriculture and Food Research Initiative Competitive Grant no. 2010-65205-20428). MLA wrote the paper and all other authors gave suggestions and comments for the improvement of paper. All authors read and approved the final manuscript. Overall coordination of the project was by JAL, JWMB, RPMAC and MAMG. All authors read and approved the final manuscript.

## Supplementary Material

Additional file 1 ** Positions of turkey genomic regions with their mapping positions and underling QTL in chicken genome.** This file contains the start and the end positions of turkey genomic regions that showed fixed haplotype for the reference alleles in domesticated populations. This file also contains information about the start and the end positions of these turkey genomic regions in chicken genome and the chicken QTL reported within these regions. Click here for file
